# Human Bone Marrow Mesenchymal Stromal Cells Attenuate Tissue Injury and Reduce Inflammation in Experimental Acute Pancreatitis

**DOI:** 10.34172/apb.2022.036

**Published:** 2021-01-31

**Authors:** Tayebeh Mahmoudi, Ali Jalili, Kamal Abdolmohammadi, Shohreh Fakhari, Fatemeh Pahlavan, Ali Shekari, Bahram Nikkhoo, Lobat Tayebi, Mohammad Reza Rahmani

**Affiliations:** ^1^Student Research Committee, Kurdistan University of Medical Sciences, Sanandaj, Iran.; ^2^Cancer and Immunology Research Center, Research Institute for Health Development, Kurdistan University of Medical Sciences, Sanandaj, Iran.; ^3^Department of Immunology, School of Medicine, Iranshahr University of Medical Sciences, Iranshahr, Iran.; ^4^Department of Basic Sciences, Farhangian University, Sanandaj, Iran.; ^5^Marquette University School of Dentistry, Milwaukee, WI, 53233, USA.

**Keywords:** Acute pancreatitis, Cerulein, Inflammation, Human bone marrow-derived mesenchymal stromal cell

## Abstract

***Purpose:*** Acute pancreatitis (AP) which is distinguished by local pancreatic necrosis, followingby systemic organ failure is known as an inflammatory disease. Up to now, there are only a fewtreatment options accessible for patients suffering from AP. In this study, we aimed to examinethe anti-inflammatory capacities of human bone marrow-derived mesenchymal stromal cells(hBM-MSCs) in a detailed AP model experiment.

***
Methods:*** AP was induced in C57BL/6 mice by intraperitoneal administration of cerulein (100μg/kg/h × 7 doses) at intervals of 1 hour. Then, 2×10^5^ MSCs were infused in the AP mice bytail vein 6 hours after the last cerulein injection. Mice were sacrificed 12 hours following theinjection of hBM-MSC, and blood samples and pancreas tissues were obtained.

***
Results:*** We first determined the presence of transplanted hBM-MSC in the pancreas of micewith AP, but not the control mice. Our data indicate that administration of hBM-MSCs to micewith AP lead to (i) decreased serum levels of amylase, lipase and myeloperoxidase activities, (ii)downregulation of proinflammatory cytokine, macrophage inflammatory protein 2 (MIP-2), and(iii) upregulation of the anti-inflammatory cytokine, interleukin 10 (IL-10). Moreover, hBM-MSCadministration results in notably attenuated cerulein-induced histopathological alternationsand edema.

***
Conclusion:*** we demonstrate that hBM-MSC attenuates AP signs and indicating that hMB-MSCtherapy could be a suitable approach for the treatment of inflammatory disease such as AP.

## Introduction

Acute pancreatitis (AP), as an important inflammatory illness, presents clinically from mild and self-limiting to severe and necrotizing disease.^1-3^ Severe AP is a major challenge for clinicians, and the mortality in this group of patients is 25-30%.^4^ Many reports have clearly revealed that unnatural activation of pancreatic enzymes initiates the majority of pathological cascades processes that finally lead to edema and histopathological changes in pancreatic tissue.^5,6^ In addition, many inflammatory cytokines, such as monocyte chemoattractant protein 1 (MCP-1), chemokine (C-X-C motif) ligand 2 (CXCL-2), interleukin 6 (IL-6) and tumor necrosis factor alpha (TNF-α) are produced by macrophages, lymphocytes and other immune cells, predominantly in pancreatic tissue. These cytokines are accountable for initiation of the inflammatory cascade that can lead to the destruction of other organs.^7,8^ Many pathological and biological aspects of AP have been recently discovered, but only a few treatment options for these patients are available. Although several remedies for the treatment of AP have been developed in past two decades, mortality has not significantly declined, indicating that development of new therapeutic options is necessary for treatment of patients with AP.^4^

Mesenchymal stromal cells (MSCs) has the capability to possess immunosuppressive and immunomodulatory properties.^9-12^ They are able to differentiate into various cells and rapidly renew themselves, making these cells a suitable candidate for regenerative medicine and tissue repair.^13-15^ Accordingly, MSCs have been utilized to cure different inflammatory diseases, such as myocardial infarction,^16^ acute kidney failure^17^ and collagen-induced arthritis.^18^ So far, several studies have determined methods of isolating MSCs from different sources, including various components of fetal tissue and adult tissue (e.g. bone marrow, adipose, and cord blood).^19^ Among the various sources of MSCs, bone marrow-derived MSCs (BM-MSCs) are regarded as the main source and most commonly applied in the clinical applications.^20,21^ Although the majority of clinical and preclinical examinations administered autologous/allogeneic transplantations, some studies utilized xenogeneic MSCs to treat various disorders in animal models.^22-24^ The purpose of this study was to investigate the anti-inflammatory effects of human bone marrow-derived (hBM)-MSCs in cerulein-induced AP in a mice model. Our data demonstrate that hBM-MSCs and their secreted factors could ameliorate the AP signs probably through their immunomodulatory and regenerative effects.

## Materials and Methods

### Animals

Thirty-two mice of the C57BL/6 strain were purchased from Pasteur Institute (Tehran, Iran) and kept in the animal house of the research center, under standardized conditions. Mice were 6-8 weeks old with average weight of 25-30 g at the time of experimentation. At least 72 hours before beginning of the experimentation, mice were carried to the laboratory to allow for acclimatization to the environment. All animal experiments were approved by the Institutional Animal Care and Research Ethics Committee of Kurdistan University of Medical Sciences, Sanandaj, Iran.

### Induction of AP and treatment procedure

Experimental AP was induced by cerulein as formerly reported.^25^ Briefly, mice were distributed into four groups (n:8 for each group) randomly. One group was injected with normal saline intraperitoneally at intervals of 1 hour (×7), which was considered the control group (Ctrl). AP group (AP), was injected cerulein (100 µg/kg, suspended in saline solution, i.p) at intervals of 1 hour (×7). Additionally, two other groups of mice were included: the Ctrl-MSC and AP-MSC, which received 2 ×10^5^ hBM–MSCs through tail vein 6 hours after the last injection of normal saline or cerulein, respectively.

### Isolation, culture and characterization of hBM-MSCs

In order to prepare hBM-MSCs, bone marrow aspirate was harvested from the iliac crest of a normal donor who was negative for HIV, HBV and HCV laboratory tests, after informed consent was obtained. Then, hBM-MSCs were cultured and isolated as previously reported.^26^ Mononuclear cells were isolated by gradient centrifugation at 2500 rpm for 30 minutes on Ficoll-Paque^TM^ Plus (Amersham Pharmacia Biotech, Uppsala, Sweden). Mononuclear cells were then plated at a concentration of 10-30 × 10^3^ cells/cm^2^ in Dulbecco’s Modified Eagle Medium (DMEM) containing 20% (v/v) of FBS. Non-adherent cells were removed 2 days later, and fresh medium was added. When the cultures reached 80-100% confluence, hBM-MSCs were trypsinized and subcultured. The purity of hBM-MSC suspensions was evaluated by flow cytometry using the following monoclonal antibodies: CD105-PE, CD45-FITC and CD90-PerCP, along with corresponding isotype controls (BD Biosciences, San Jose, CA, USA). Finally, the suspensions were analyzed utilizing a BD FACS Canto^TM^ flow cytometry System (BD Biosciences, CA).

### Transplantation of hBM–MSCs to experimental models

Human BM-MSC administration was performed under anesthesia (ketamine 100 mg/kg + xylazine 10 mg/kg, i.p). Mice received 2×10^5^ hBM–MSCs through tail vein by insulin syringes (28 gauge) 6 hours after the last normal saline (Ctrl-MSC) or cerulein (AP-MSC) injection. 12 hours after injection of hBM-MSCs the mice were scarified by cervical dislocation after anesthesia (ketamine 100 mg/kg + xylazine 10 mg/kg, i.p), and the bloods (500-1000 µL) were collected by a direct intracardiac puncture.

Pancreatic tissue was removed promptly and shared in several parts, either fixed in formalin for histopathological examinations or frozen at -70°C for polymerase chain reaction (PCR) analysis.

### Detection of hBM–MSCs in pancreas tissues

The PCR assay was performed to detect the presence of transplanted hBM-MSC in the pancreatic tissue of mice.^23^ DNA was extracted from 20 mg of pancreatic tissue using Prime Prep Genomic DNA Isolation Kit (Genet Bio Kit) according to the manufacturer’s instructions. A pair of human-specific primers (forward: 5-AGCCACTTTCCACACAGAC-3, reverse: 5-AGTAGTATGGGAGTGGGAG-3) describing a 219 bp region of cytochrome B mitochondrial gen was designed by Gen Runner software. PCR reaction was performed with 2X PCR master mix and included an initial step of denaturation at the temperature of 95°C for the duration of 10 minutes followed by 35 cycles (95°C for 30 seconds, 54°C for 35 seconds, 72°C for 5 minutes. The PCR products were isolated by an agarose gel electrophoresis and stained with ethidium bromide.

### Determination of pancreatic edema and serum amylase and lipase levels

The fresh pancreatic tissue was collected from each group of mice, and pancreatic edema was determined as previously reported.^27^ Briefly, pancreas samples were weighed on aluminum foil using an electronic balance, dried at 95°C for 12 hours and reweighed. The water content of the pancreatic tissue was evaluated as the variation between wet and dry tissue weight and described as a percent of wet tissue weight. The serum amylase and lipase were assayed using an automatic biochemistry analyzer (BT3000, Rome, Italy) in the clinical chemistry laboratory (Behsat hospital, Sanandaj, Iran).

### Histopathological examination

Formaldehyde-fixed (10% buffered formaldehyde) pancreatic tissue was embedded in paraffin, sectioned (5 µm) and stained using hematoxylin-eosin (H&E) to evaluate infiltration of inflammatory cells and acinar cell injury or necrosis. Ten randomly selected sections for each tissue sample were analyzed after staining. Histological evaluation of pancreatic sections was performed by pathologist collogues (B.N), who was blinded to each group.

### Estimation of MPO enzyme activity

Neutrophil sequestration within the pancreatic tissue was evaluated by assessing the tissue myeloperoxidase (MPO) activity.^28^ Tissue samples initially had frozen at -70°C and were thawed at 4°C. The tissues were then homogenized in 20 mmol/L phosphate buffer (PH 7.4) and centrifuged (13 000 × g, 10 minutes, 4°C). The consequential pellet was resuspended with 0.5% hexadecyl trimethyl ammonium bromide (Sigma-Aldrich) in 50 mmol/L phosphate buffer (PH 6.0). The suspension was exposed to four freeze-thaw cycles and further sonicated (40 seconds on ice). The sample was again centrifuged (13 000 × g, 8 minutes, 4°C). MPO activity in the obtained supernatant was determined using TMB liquid substrate system for ELISA at 405 nm with the Tecan spectrophotometer plate reader. The results were corrected for the absorbance per mg of tissue weight (A°/mg) in the respective samples and expressed as an increase over the control group.

### Analysis of messenger RNA expression by reverse transcription PCR

Finally, determination of the expression of inflammatory cytokines in pancreatic tissue was assessed using by RT-PCR. RNA was isolated utilizing RNA extraction kit (Bioflux, Basel, Switzerland) and RNA was transcripted into cDNA utilizing of Bioneer kit (Bioneer, Daejeon, South Korea). Amplification was conducted in a thermocycler (Mastercycler, Eppendorf, Westbury, NY). The PCR profile consisted of an initial denaturation for 5 min (94°C), followed by 40 cycles for 30 seconds (95°C) and annealing temperature for each gene (30 seconds) and the final step for 45 seconds (72°C). The PCR produces were electrophoresed on a 2% agarose gel comprising ethidium bromide. ImageJ software was employed for the densitometry analysis of the gel bands. The following primers were used: mouse MIP-2 (forward: 5-TCATAGCCACTCTCAAGGG-3, reverse: 5-TTGGTTCTTCCGTTGAGGG-3) and mouse IL-10 (forward: 5-GCGCTGTCATCGATTTCTC-3, reverse: 5-CCGTTAGCTAAGATCCCTG-3). Mouse ß actin (forward: 5-CTTGGGTATGGAATCCTGTG-3, reverse: 5-ACTGTGTTGGCATAGAGGTC-3) was used as a housekeeping gene.

### Statistical analysis

Numeral data are displayed as mean ± SD of 8 mice. One-way ANOVA and Tukey post hoc test was utilized to determine the difference between groups and *P* < 0.05 demonstrated statistical significance.

## Results

### Characterization of hBM-MSCs

**Figure 1:**
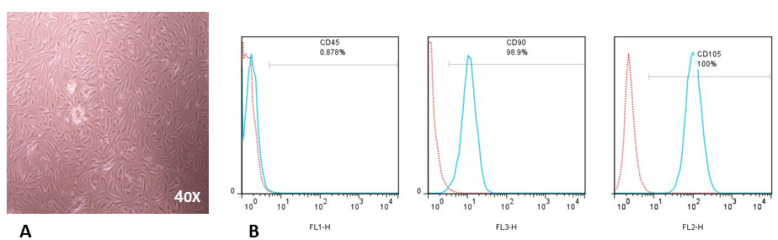
**
Characterization of hBM-MSCs.** (A) Morphological evaluation (X40) of hBM-MSCs in passage four. (B) Representative FACS analysis indicates that hBM-MSCs are negative for hematopoietic markers (CD45) but positive for MSC markers (CD90 and CD105). The red histogram illustrates isotype control antibody and turquoise histograms demonstrate CD45, CD90, or CD105 markers. hBM-MSC were stained with anti-CD105-PE, anti-CD45-FITC, and anti-CD90-PerCP for 45 min at 4ºC and ran by FACS Calibur (BD, CA).

To isolate hBM-MSCs, we discarded the non-adherent cells, and cultured the adherent cells as described in the previous section. After 3-4 days of culture, hBM–MSCs showed features distinct from other adherent cells (adherent hematopoietic cells and monocytes). They were spindle-shaped cells or fibroblast-like cells and had morphology resembling epithelial cells (Figure 1A). In the fourth passage, hBM-MSCs were inspected for the expression of specific surface markers by flow cytometry. Our data demonstrates that hBM-MSCs were positive for CD90 (98.9%), CD105 (100%) and negative for CD45 (0.87%) (Figure 1B).

### Intravenously-infused hBM-MSCs determined in pancreatic tissue of mice with AP

**Figure 2:**
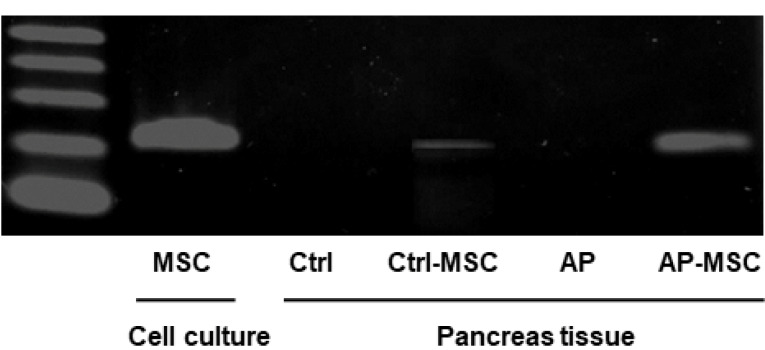
**
Determination of human Cytochrome B DNA.** PCR results confirm the existence of the human cytochrome B gene in pancreatic tissue after the infusion of hBM-MSC. Human cytochrome B gene was detected in the pancreas of 4 groups, as described in the materials & methods section. In vitro cultured hBM-MSC at passage four was used as the positive control.

Next, in order to examine the presence of hBM-MSCs in pancreatic tissue, PCR assay was employed to detect human cytochrome B gene. As shown in Figure 2, human DNA was more strongly detected in the AP-MSC group than the other groups, particularly, Ctrl-MSC group with a normal pancreas tissue. The hBM-MSCs cultured in vitro were used as positive control.

### Effect of hBM–MSCs on pancreatitis indications

**Figure 3:**
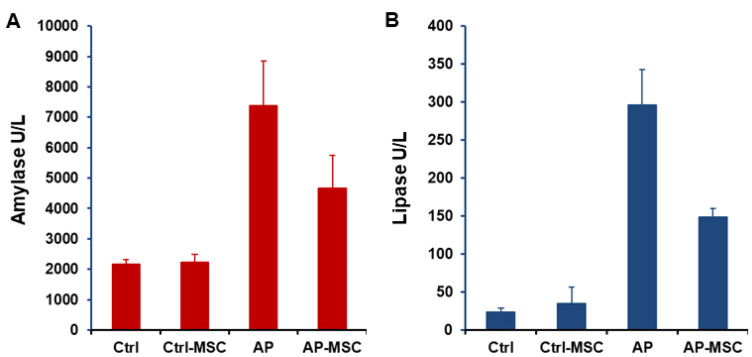
**
The effects of hBM-MSCs on amylase and lipase activities.** Blood samples were collected from mice 12 h after administration of hBM-MSCs. Infusion of hBM-MSCs considerably lowers the rise of amylase (A) and lipase (B) induced by cerulein in the AP group. Each value represents the mean ± SEM for 6-8 mice, and levels were known significant at *P* < 0.05.

**Figure 4:**
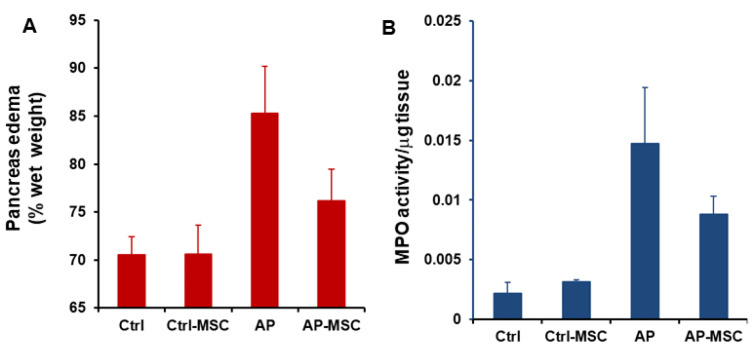
**
Effects of hBM-MSCs on pancreatic edema and myeloperoxidase activity in pancreatic tissue.** (A) The administration of hBM-MSCs significantly reduces pancreatic edema induced by cerulein. (B) hBM-MSCs treatment reduces MPO activity in mice with AP. The results were corrected for the absorbance per mg of tissue weight (OD/mg) in the respective samples. Each value is the mean ± SEM for 6-8 mice and levels were considered significant at *P* < 0.05.

In this study, the severity of cerulein-induced AP was distinguished by elevated levels of both amylase and lipase in the serum of mice. Also, levels of these enzymes were evaluated after treatment with hBM-MSCs. As presented in the Figure 3A and 3B, levels of both amylase and lipase are meaningfully reduced in the serum of treated mice. The water content of pancreatic tissue was quantified to determinate pancreatic edema. Data shows that edema is considerably raised in the AP group in comparison with the control group, however in when hBM-MSCs-treated mice, pancreatic edema was significantly decreased (Figure 4A).

### MPO activity and pancreas histology after infusion of hBM-MSCs

**Figure 5:**
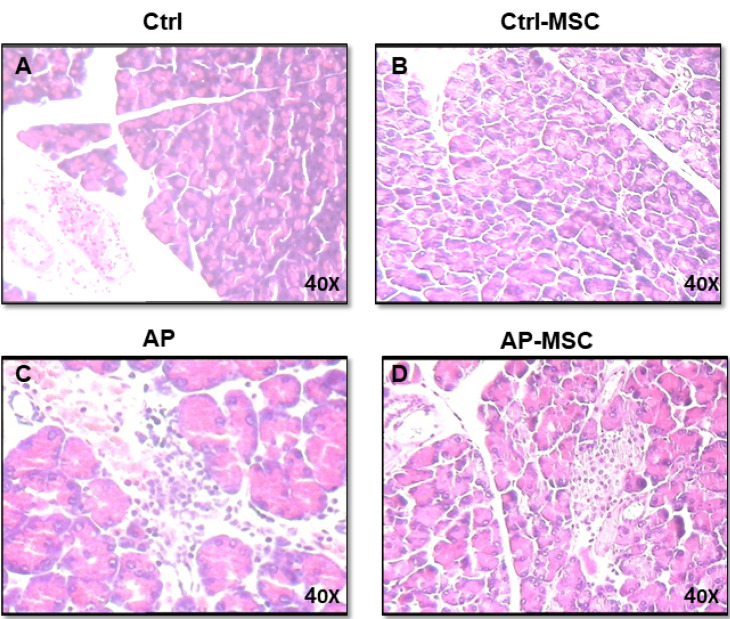
**
Effects of hBM-MSCs on cerulein-induced histological alternations of the pancreas.** (A) Demonstrates that the histological characters of the pancreas obtained from control mice. (B) Histological features of pancreas tissues from control mice that had been treated with hBM-MSCs (Ctrl+MSCs) show no significant difference. (C) Meanwhile, pancreatic sections of cerulein-induced acute pancreatitis mice exhibit tissue injury characterized by interstitial edema, inflammatory cell infiltration, and acinar cell necrosis. (D) The pancreatic section from AP mice that had been treated with hBM-MSCs (AP+MSCs) shows fewer histological alterations compared with the AP group. All experiments have been performed six times for each group (one mouse per group/per time point). The figure (X40) is the representative of the six experiments.

The MPO activity was evaluated to quantify the extent of neutrophil and monocyte accumulation in pancreatic tissue and demonstrate the extent of inflammatory cells infiltration in tissue. As shown in Figure 4B, MPO activities are expressively increased in AP mice in comparison with the control group. However, infusion of hBM-MSCs to the AP mice results in a substantial decrease of MPO activity in comparison with the AP group. In addition, histological examination of sections of the pancreatic tissue shows evident tissue damage with necrosis of acinar cells and penetration of inflammatory cells in the mice with AP. However, these histopathological alternations were restored after treatment with hBM-MSCs (Figure 5).

### Effect of hBM–MSCs on pancreatic inflammatory cytokines

**Figure 6:**
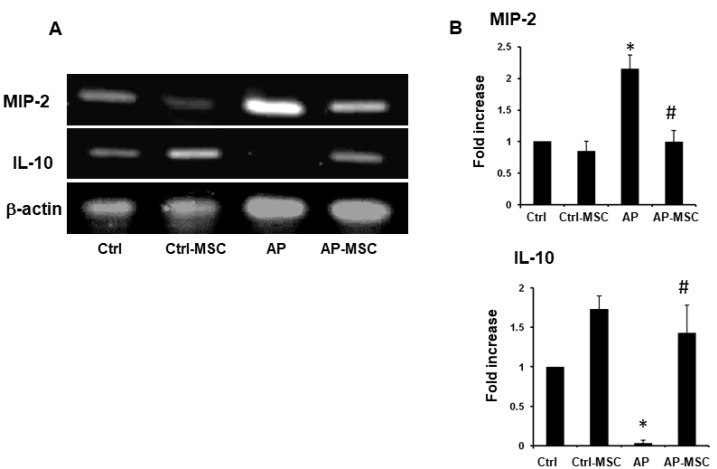
**
The effect of hBM-MSCs on expression MIP-2 and IL-10 expression in pancreatic tissue **. mRNA was isolated and MIP-2 and IL-10 expression was tested using RT-PCR. (A) RT-PCR analysis of mRNA transcripts for MIP-2 and IL-10 in the pancreas of the control, AP, and AP-MSC mice. β-actin was employed as an internal loading control for the RT-PCR. (B) Expression of MIP-2 and IL-10 in the four groups of mice were semi-quantified using ImageJ software. Each value represents the mean ± SEM of densitometer bands of pooled data of at least six mice are shown.

As inflammation and its players, such as proinflammatory cytokines, associate to the pathobiology of AP, we inspected the effect of hBM-MSCs on the expression levels of two important mediators in pancreas tissue. We observed that the expression of MIP-2 was upregulated in mice with AP, but its expression was significantly decreased in the group that was treated with hBM-MSCs (Figure 6). In contrast, the anti-inflammatory cytokine IL-10 expression is significantly reduced in the AP group, whereas hBM–MSCs considerably enhance its expression in the AP-MSC group.

## Discussion

Although a number of biological and pathological aspects of AP as an inflammatory disease have been recently discovered, and many therapeutic approaches have been offered to the patients, the full recovery in 25-30% of patients with severe AP not yet been evaluated.^4,29^ However, recent findings indicate that MSCs, due to their immunomodulatory capacity, could be a suitable candidate for immunotherapy of many inflammatory disorders, including AP. In this study, we investigated the anti-inflammatory effects of hBM–MSCs on an AP model of mice and observed that transplanted MSCs significantly decrease enzymatic activities of serum amylase and lipase, as well as decreasing other pancreatitis indications, including edema, MPO activity, and pancreatic pathological changes.

Mounting evidence indicates that MSC migrates to the injured tissue to either repair or reduce the inflammatory cascade. To participate in tissue repair, MSC needs to cooperate and interact closely with stromal and inflammatory cells, implying that MSC should migrate from circulation or other sources toward the damaged tissue.^30^ Moreover, we have recently shown that intratracheally administration of human umbilical cord vein MSCs results in attenuation of pulmonary fibrosis in a mouse model.^23^ Although the routes of administration in our previous and current work were different, the MSCs still exhibited immunomodulatory activity. Herein, we used human cytochrome B DNA as a marker to determine the presence of hBM-MSCs in the pancreas of mice with AP after treatment with these cells and found that the amount of xenogeneic genome was much higher in the AP mice than the control group, indicating that hBM-MSCs migrated to the inflamed tissue of the pancreas. Since MSCs exhibit immunomodulatory properties, they are able to evade immune system recognition. Moreover, they possess a very low level of MHC-class I, implying that hBM-MSC not only could not be recognized by the exogenic host but also may mediate immunomodulatory activities.^31^ It has also been reported by Jung et al that the severity of tissue injury can influence both migration and function of MSCs after transplantation,^32^ demonstrating that injured tissues may chemoattract MSCs to the site of inflammation.

The migration process of MSCs is mainly dependent on chemokine attraction. A key player in this process is stromal-derived factor (SDF-1), and via binding to its receptor, CXCR4 has a significant role in the migration of MSCs.^26^ Gong et al showed that SDF-1 is upregulated in the pancreatic tissue of rats with induced-AP, and the SDF-1/CXCR4 axis regulates the trafficking of MSC towards the pancreas as the migration of these cells was blocked by anti-CXCR4 antibody or the CXCR4 inhibitor (AMD3100).^33^ In addition, another report has exhibited that CXCR4 is expressed at high levels in the bone marrow and ischemic tissues but disappears from MSC surface after cultivation. However, hypoxia and proinflammatory cytokines can restore CXCR4 levels.^34^ Taken together, we postulate that increased levels of many pro-inflammatory cytokines and hypoxia-induced state in the injured pancreas could upregulate both SDF-1 and CXCR4, resulting in hBM-MSC leaving circulation to enter the inflamed pancreas.

AP is the outcome of inopportune activation of proenzymes within acinar cells and following this process, the levels of digestive enzymes immediately increase in patient serum. We administered experimental AP by cerulein injection, which is a typical animal model of AP that induces pancreatitis signs similar to AP disease in human.^3,35^ Data collected for this study demonstrate that hBM–MSCs treatment results in a reduction of serum amylase and decrease of lipase levels, as well as pathological alternations of AP, indicating that hBM-MSCs have beneficial effects on experimental models. Accordingly, Yang et al showed that umbilical cord MSCs alleviated severe AP disease in a rat model in a time-dependent and dose-dependent manner. They proposed that earlier utilization of MSCs, with appropriate doses after induction of severe AP, more effectively helps alleviate the consequence of pancreatitis. Since transplanted MSCs cannot differentiate into other cells in a short time span, the hypothesis has emerged that MSCs regulate immune responses through paracrine effects.^36^ In accordance with the results obtained in the current study, others have previously reported that the administration of MSC attenuated AP in animal models.^24,37-39^ AP was induced by sodium taurocholate in rat models in most of those studies, however, our study is the first study using hBM-MSCs to treat cerulean-induced AP in mice model. Data from our laboratory confirm these notions that, at least in the case of AP, hBM-MSCs cannot differentiate into other pancreatic cell types and more likely attenuate the AP symptoms and findings by decreasing inflammatory cascades in the pancreas. We have observed that the infusion of 2×105 cells of hBM-MSCs 6 hours after AP induction significantly decreases lipase, amylase, and MPO activity, in addition to creating histopathological alterations. However, injection of hBM-MSCs at 12 hours and 24 hours after AP induction did not display a significant effect (data was not shown). So far, we propose that hBM–MSCs can have a beneficial influence in the treatment of AP if these cells are utilized at the optimal time. Based on the fact that the majority of AP in human are self-limited in nature, cellular therapy using hBM–MSCs would not be a therapeutic option.^37^ However, either hBM–MSCs or their derived mediators could be suitable approaches for the treatment of severe AP and autoimmune AP.

Mounting evidence from previous reports implies that the accumulation of neutrophils into pancreatic tissue is governed by the interaction of chemokines and their receptors.^40,41^ MIP-2 is a robust chemoattractant for neutrophils in mice and participates in leukocyte trafficking as the migration of neutrophils reduced in MIP-2-/- mice.^42,43^ By employing flow cytometry analysis, we previously demonstrated that the number of pancreatic neutrophils is remarkably increased in mice with AP, which is associated with the levels of MIP-2.^27^ Accordingly, we exhibit that the expression of MIP-2 is considerably enhanced in the cerulein-induced AP, confirming the previous study.^27^ However, the administration of hBM-MSCs into the tail vein of the mice with AP resulted in a considerable reduction in the expression of the inflammatory MIP-2 in pancreatic tissue. IL-10 is known to have an important role in the balance of immune function and it has been stated that MSC accelerates tissue repair in experimental models by enhancing the IL-10 levels.^44^ Interestingly, our data show that the expression of IL-10 at mRNA levels was downregulated in AP mice, then upregulated after treatment with hBM-MSC. This is in agreement with a recent report, showing that the endoscopic administration of MSCs reduces inflammation in experimental colitis.^45^ Based on our data and previous observations, we envision that hBM-MSCs may restore the imbalance of pro-inflammatory/anti-inflammatory cytokines during AP.

## Conclusion

This study demonstrates that hBM-MSCs are able to inhibit inflammatory reactions and improve damaged tissue of the pancreas in mice models of AP disease. Although this work confirms that cell therapy using hBM-MSCs at the optimal time could be a functional approach to treat AP mice, further investigation employing physically larger animals is required before making any clinical conclusion for humans. Moreover, understanding new mechanisms of hBM-MSC effects in pancreatitis and investigation of MSCT progress in AP remains for the further relevant study.

## Acknowledgments

A grant (grant no. 14/10693) from Kurdistan University of Medical Sciences (KUMS) was offered to AJ. TM was an MSc student in medical immunology at KUMS.

## Ethical Issues

All animal experiments were approved by the Ethics Committee of Kurdistan University of Medical Sciences, Sanandaj, Iran (Project number: 14/10693, Approval date: June 25, 2013).

## Conflict of Interest

The authors declare that there is no competing financial interest.
